# Investigating the role of lncRNA SNHG14 in early diagnosis and prognosis of acute pancreatitis: a bioinformatics exploration

**DOI:** 10.1186/s41065-026-00656-z

**Published:** 2026-03-11

**Authors:** Qilin Zhu, Bin Liang, Dongqin Shen, Wei Chen, Hanqing Liao

**Affiliations:** 1 Department of Gastroenterology, The Affiliated Suzhou Hosptal of Nanjing University Medical School, Suzhou City, 215000 China; 2 Department of Breast, Zhangjiakou First Hospital, Zhangjiakou City, 075000 China; 3 ICU, Jiangsu Provincial Hospital of Chinese Medicine Chongqing Hospital (Chongqing Yongchuan District Traditional Chinese Medicine Hospital), Chongqing, 402160 China; 4 Department of Critical Care Medicine, The First People’s Hospital of Lanzhou, No.1 Wujiayuan West Street, Qilihe District, Lanzhou City, 730050 Gansu Province China; 5 ICU, Ji’an Central People’s Hospital, No.80, Ji’an South Avenue, Jizhou District, Ji’an City, 343000 Jiangxi Province China

**Keywords:** Acute pancreatitis, SNHG14, miR-30a-5p, Outcome, Inflammation

## Abstract

**Background:**

Acute pancreatitis (AP) is a severe inflammatory disease. Early and accurate assessment of disease severity remains intricate, highlighting the need for novel biomarkers. Long non-coding RNAs (lncRNAs) play crucial roles in AP pathogenesis.

**Objective:**

This study aimed to investigate the clinical significance and mechanism functional of SNHG14 in AP.

**Methods:**

A prospective cohort of 307 participants (148 AP patients, 159 controls) was recruited. Serum SNHG14 and miR-30a-5p levels were detected by qRT-PCR. An in vitro AP model was established using cerulein (100 nM, 24 h) treatment in AR42J and HPDE6-C7 cells. Functional assays (CCK-8, flow cytometry, ELISA) were performed following SNHG14 knockdown. Bioinformatics analysis was employed for target prediction (starBase, TargetScan, miRDB) and pathway enrichment (KEGG/GO). The SNHG14/miR-30a-5p interaction was confirmed by dual-luciferase assay.

**Results:**

SNHG14 was significantly up-regulated in AP patients and correlated with disease severity. It showed diagnostic potential for AP (AUC = 0.835) and for identifying severe AP (AUC = 0.757). High SNHG14 level was an independent predictor of poor 28-day prognosis (HR = 4.31, *P* = 0.018). In vitro, SNHG14 knockdown alleviated cerulein-induced cell apoptosis and secretion of TNF-α, IL-10, and IL-6. SNHG14 functioned as a sponger for miR-30a-5p, and its effects were reversed by miR-30a-5p inhibition. Bioinformatic analysis showed that miR-30a-5p target genes are enriched in autophagy, ubiquitin-mediated proteolysis, and inflammatory pathways.

**Conclusions:**

SNHG14 is a promising biomarker for AP severity and prognosis. The SNHG14/miR-30a-5p axis plays a critical role in regulating inflammation and apoptosis in AP.

**Significance:**

This study is the first to delineate the clinical and mechanistic role of the SNHG14/miR-30a-5p axis in AP. It provides a potential candidate for non-invasive RNA-based diagnostic strategies and identifies a potential molecular target for intervention of AP. However, it represents an auxiliary molecular therapy strategies rather than a replacement for existing management.

**Supplementary Information:**

The online version contains supplementary material available at 10.1186/s41065-026-00656-z.

## Background

Acute pancreatitis (AP) is a prevalent acute digestive system condition with varying clinical severity [1, 2]. Severe cases may progress to severe AP (SAP), characterized by persistent organ dysfunction, systemic inflammatory response syndrome, and high mortality rates (up to 30%) [3–5]. The pathogenesis of AP is complex [6–11], involving multiple pathways including abnormal activation of pancreatic enzymes, release of inflammatory mediators, dysregulated autophagy, oxidative stress, and apoptosis. Currently, AP diagnosis relies primarily on clinical symptoms, serum amylase and lipase levels, and imaging findings [12, 13]. Recent studies have shown that modifications to proteins, such as acetylation, can influence how the body responds to stress [14]. Research into natural bioactive compounds continues to provide inspiration for anti-inflammatory therapies like AP [15, 16]. However, accurately assessing disease severity and predicting prognosis during early stages remains challenging. Developing of non-invasive, dynamic monitoring biomarkers [17] requires deeper exploration of disease-specific molecular mechanisms and integration with innovative diagnostic and therapeutic modalities [18].

Long non-coding RNAs (lncRNAs) are a class of promising molecules that have been found to exert significant regulatory roles in pancreatitis. Small Nucleolar RNA Host Gene 14 (SNHG14) is located on the forward strand of chr 15, extending in the reverse direction to the region housing the ubiquitin protein ligase E3A gene (UBE3A), thereby regulating UBE3A expression [19]. A systematic literature search was conducted across PubMed, Web of Science, Scopus, and SinoMed databases (2000–2024) to assess the current landscape. The keywords for search focused on “lncRNA”, “SNHG14”, “acute pancreatitis”, and “miR-30a-5p”. SNHG14 was selected as a candidate due to its emerging undefined role in systemic inflammatory conditions, distinct from other well-studied AP-associated lncRNAs. SNHG14 is expressed in various tissues [20] and is overexpressed in pancreatic cancer tumors. SNHG14 promotes gemcitabine resistance for pancreatic cancer through miR-101/autophagy axis [21], suggesting its potential significance in pancreatic diseases. Besides, SNHG14 has been implicated in lipopolysaccharide-induced acute kidney injury through the autophagy pathway [22]. This cross-tissue inflammatory signature identifies SNHG14 as a conserved amplifier of cytokine storms, critical to systemic inflammatory response in AP. Notably, preliminary bioinformatics screening showed that SNHG14 target miR-30a-5p is enriched in key AP pathobiology pathways related to inflammation and autophagy. miR-30a-5p participates in regulating autophagy, inflammation, and apoptosis processes across multiple diseases, including AP [23, 24]. LncRNAs are well-recognized AP biomarkers, PVT1 primarily regulates local autophagy [23]. A critical gap remains in identifying regulators linking local pancreatic injury to SAP-related systemic inflammation (the hallmark of SAP). SNHG14 is uniquely suited to fill this gap due to its genomic and functional features [25], especially by targeting miR-30a-5p. Although SNHG14/miR-30a-5p axis has been reported in podocyte injury, its role in AP has not been elucidated [26].

This study integrates clinical effects with mechanistic exploration to investigate the diagnostic and prognostic potential and molecular mechanism of SNHG14 in AP. Serum SNHG14 was first detected in AP patients to evaluate its diagnostic potential. Functional validation was subsequently performed using cell models. Finally, bioinformatic analyses were conducted to elucidate the mechanism by which SNHG14 regulates downstream networks via miR-30a-5p, thus establishing a complete clinical-to-mechanistic evidence chain.

## Methods

### Sample calculation and ethical approval

A prior power analysis using G*Power 3.1 software determined the minimum sample size needed to identify a significant difference between two independent groups. The analysis used a two-tailed t-test with the following parameters: effect size of 0.5, α of 0.05, and statistical power of 0.95. This yielded a required total of 210 participants across both groups. This research was conducted in accordance with the ethical guidelines of the Declaration of Helsinki and received approval from the Institutional Review Board of The First People’s Hospital of Lanzhou. All patients and healthy volunteers provided written informed consent before enrollment. Participants received clear explanations of all procedures and assurance that their personal and medical information would remain confidential.

### Study subjects

AP patients and healthy controls were collected from The First People’s Hospital of Lanzhou from January 2023 to December 2024. AP was diagnosed using the 2012 Atlanta classification criteria [27], which require at least two of the following conditions: (1) severe, acute epigastric pain that frequently spreads to the back; (2) serum amylase and/or lipase levels at least three times the upper limit of normal; (3) characteristic AP findings on contrast-enhanced CT, MRI, or abdominal ultrasound. All participants were at least 18 years old and underwent hospital admission with initial blood collection within 24 h of symptom onset. Individuals meeting the following exclusion criteria were not considered for this research: (1) acute exacerbation of chronic pancreatitis; (2) AP caused by pancreatic or periampullary tumors; (3) history of pancreatic surgery or trauma; (4) severe primary cardiac, hepatic, renal, pulmonary, or hematological diseases; (5) known autoimmune diseases, active infections (such as HIV or viral hepatitis), or long-term immunosuppressants use; (6) cancer; (7) pregnancy or lactation; (8) incomplete clinical data preventing accurate severity grading or prognosis assessment; (9) death or discharge within 24 h of admission; (10) incomplete data. AP severity was classified according to the 2012 Atlanta criteria into mild, moderate, and severe AP. Healthy controls were selected from the health examination center of the same hospital, with all routine tests within normal ranges. Controls were matched 1:1 with AP patients by age (± 3 years) and gender through consecutive enrollment.

### Data collection

Clinical data and prognosis were recorded. The prognostic endpoint was set at 28 days after admission. Prognostic assessment adopted a composite endpoint, defined as the earliest occurrence of any of the following events within the 28-day observation period: (1) Secondary pancreatic infection confirmed by computed tomography (CT) - guided aspiration and microbial culture; (2) New or persistent organ failure (≥ 48 h) determined by modified Marshall score ≥ 2; or (3) All-cause mortality. Patients who met any event in the composite endpoint were classified into the poor prognosis group, with the event occurrence date recorded as the event time. Patients without any of the above events during the 28-days were classified as the favorable prognosis group.

### Enzyme-Linked Immunosorbent Assay (ELISA)

Serum levels of multiple indicators were measured using an automated biochemical and immunoassay analyzer. These included lipase (LIPA) (Lipase Assay kit, ab102524, Abcam, Cambridge, UK), amylase (AMY) (Amylase Assay Kit, BC0615, Solarbio, Beijing, China), hematocrit (HCT), total cholesterol (TC) (ml063717), triglycerides (TG) (TG Content Assay Kit, BC0625, Solarbio, Beijing, China), C-reactive protein (CRP) (ml057570), procalcitonin (PCT) (ml106700), tumor necrosis factor-alpha (TNF-α) (ml106471), interleukin (IL)-10 (ml064299), and IL-6 (ml058097). All assays were performed according to the manufacturer’s instructions to ensure accuracy and reliability. All experiments were replicated five times. For each experiment, kit-supplied standards were run in parallel, and the correlation coefficient (R²) of each curve exceeded 0.99. All ELISA kits were bought from Shanghai Enzyme-linked Biotechnology Co., Ltd.

### RNA Extraction and Quantitative Real-Time PCR (qRT-PCR)

Total RNA was isolated from serum samples utilizing TRIzol reagent (15596018CN, Invitrogen). RNA quality and integrity were evaluated by agarose gel electrophoresis. The expression levels of SNHG14 and miR-30a-5p were quantified via qRT-PCR using the HiScript II One Step RT-PCR Kit (P611-01, Vazyme, Nanjing, China). Internal references included GAPDH and U6. Relative expression was calculated using the 2^-ΔΔCt^ method. Primer sequences are provided in Supplementary Table 1.

### Cell culture and cell model construction

Rat pancreatic exocrine cells (AR42J, HTX1950, Otwo Biotech, Shenzhen, China) and human pancreatic duct epithelial cells (HPDE6-C7, HTX1979C, Otwo Biotech, Shenzhen, China) were maintained in DMEM-H medium supplemented with 10% fetal bovine serum (FBS) and 1% penicillin-streptomycin. Cells were plated in 6-well plates and cultivated until they reached approximately 80% confluence. To create the AP cell model, cells (1 × 10^5^ cells/mL) were seeded in 6-well plate and exposed to 100 nM cerulein diluted in phosphate buffer solution (PBS) for 24 h as described in a previous study [28]. The control group received PBS treatment only.

### Cell transfection

Lentiviral vectors were constructed and transfected into AR42J and HPDE6-C7 cells using Lipofectamine 3.0 (L3000008, Invitrogen, Thermo Fisher Scientific Inc.). Lentiviral supernatant was collected at 48 h and used for cell infection. Cells were infected with the supernatant at a multiplicity of infection (MOI) of 20 in medium supplemented with 8 µg/mL Polybrene. The medium was replaced with complete medium 8–12 h post-infection. Stable cell lines were established through selection with 1 µg/mL puromycin (60209ES10, Yeasen, Shanghai, China) for 5–7 days. The knockdown efficiency of SNHG14 was verified via qRT-PCR. The si-SNHG14 and its negative control (NC) were synthesized by GeneChem (Shanghai, China). The miR-30a-5p inhibitor and its control inhibitor were obtained from MedChemExpress (Shanghai, China). These sequences are shown in Supplementary Table 1.

### Cell viability and apoptosis assays

Cell viability was assessed using the cell counting kit (CCK)-8 assay (ml091654, Shanghai Enzyme-linked Biotechnology Co., Ltd.). A total of 1 × 10⁴ cells were plated in each well of 96-well plates. After 24 h of culture, CCK-8 solution was added and incubated for 2 h, and absorbance was measured at 450 nm. Cell apoptosis was analyzed by flow cytometry 24 h after stable transfection, employing the Annexin V-FITC/PI Apoptosis Detection Kit (40302ES60, Yeasen, Shanghai, China).

### Dual-luciferase reporter assay

The potential target genes of SNHG14 were identified through the starBase v3.0 database. The validation of the interaction between SNHG14 and miR-30a-5p was conducted using a dual-luciferase reporter assay. To confirm this interaction, luciferase reporter vectors that contained both the wild-type (wt) and mutant (mut) sequences of SNHG14 were utilized. These vectors were co-transfected with either miR-30a-5p inhibitors or a NC inhibitor into AR42J and HPDE6-C7 cells. Following a 48-hour transfection period, the measurement of luciferase activity was carried out using the Dual-Luciferase Reporter Assay System (Promega). Transfection efficiency was normalized as the ratio of Firefly to Renilla luciferase activity. Each experiment was conducted in quintuplicate, and relative luciferase activity was determined by calculating the ratio of experimental group activity to NC group activity.

### Target gene selection of miR-30a-5p

Target genes for miR-30a-5p were identified using three databases ENCORI v3.0, TargetScanHuman 8.0, and miRDB v6.0 databases based on specific criteria: for miRDB, a Target Score greater than 90; for TargetScan, a total context + + score below − 0.30; and for ENCORI, a CLIP-DATA count exceeding 1. Protein-protein interaction (PPI) network of the overlapping target genes was analyzed by STRING v12.0, with the interaction confidence set to 0.4 and a false discovery rate (FDR) threshold of < 0.5.

Gene Ontology (GO) function and Kyoto Encyclopedia of Genes and Genomes (KEGG) pathway enrichment of the overlapping target genes were then analyzed using an online tool, SRplot [29] (http://bioinformatics.com.cn/?keywords=pathway), with |log2 fold change (FC)|>1.0, FDR < 0.05.

### Statistical analysis

Data are expressed as mean ± standard deviation. Group differences were examined using Student’s t-test or a non-parametric method. Diagnostic and prognostic values were assessed using receiver operating characteristic (ROC) curves and Kaplan-Meier (KM) survival analysis. DeLong’s test was utilized to compare the area under the ROC curves (AUCs) between different diagnostic indicators in SPSS 22.0. Multivariate logistic regression was utilized to identify independent predictors of SAP. Cox regression analysis was performed to determine risk factors associated with poor prognosis, presented as hazard ratio (HR) with 95% confidence interval (95% CI). Given the limited number of poor outcomes (*n* = 29), a two-stage modeling strategy was adopted to decrease the overfitting risk in the multivariate Cox regression. Variables with *P* < 0.05 in univariate analyses were included in the initial multivariate model. Then, least absolute shrinkage and selection operator (LASSO) regression was used for variable selection. Only LASSO-screened variables were included in the final multivariate Cox model. All continuous predictors were standardized before model fitting. Multicollinearity assessment was assessed using the variance inflation factor (VIF). Variables with VIF > 5 were removed. Spearman’s rank correlation coefficient was used to evaluate correlations. The significance threshold was set at 0.05. Individual data points were presented in all column/box plots. Exact sample sizes are shown in figure legends.

## Results

### Clinical features of AP patients

This study collected 148 AP cases and 159 controls, with a statistical power exceeding 0.99. No significant differences in age, gender, or BMI were observed between the two groups (Table 1, *P* > 0.05), suggesting strong comparability. AP cases exhibited significantly elevated levels of serum TG, WBC, LIPA, AMY, CRP, PCT, TNF-α, IL-10, and IL-6 in comparison to the healthy controls (*P* < 0.05). Furthermore, the TC level was notably lower in the AP group than in the control group (*P* = 0.003). Among AP patients, the predominant cause was biliary factors (53 cases), followed by alcohol consumption (30 cases) and hyperlipidemia (28 cases). Based on the 2012 Atlanta classification, patients were categorized as mild (41 cases), moderate (72 cases), or severe (35 cases). A total of 29 patients achieved a poor composite endpoint; the median time-to-first event was 5 days (interquartile range [IQR] 3–8 days). Among them, 6 had secondary infections (5 [4–7] days), 21 had organ failure (3 [2–6] days), and 9 died (7 [4–12] days). Notably, 5 patients manifested both organ failure and subsequent death, with the organ failure event serving as the time-to-event in composite analysis.

**Table 1:** Clinical features of acute pancreatitis patients

Age	Control group (*n* = 159)	AP group (*n* = 148)	*P*
	49.89 ± 12.04	49.53 ± 11.92	0.789
Gender (male/female)	87/72	83/65	0.810
BMI (kg/m^2^)	22.64 ± 2.25	22.88 ± 2.52	0.370
TC (mmol/L)	4.60 ± 0.64	4.26 ± 1.30	0.003
TG (mmol/L)	1.65 (1.25–1.97)	1.97 (1.15–2.89)	< 0.001^*^
WBC (×10^9^/L)	6.08 ± 2.83	11.93 ± 2.98	< 0.001
HCT (%)	42.09 ± 2.80	41.76 ± 2.14	0.258
LIPA (U/L)	77.16 ± 42.64	296.79 ± 110.80	< 0.001
AMY (U/L)	68.00 (48.00-88.50)	1110.74 (496.67-1618.11)	< 0.001^*^
CRP (ng/mL)	5.15 ± 2.84	150.08 ± 63.33	< 0.001
PCT (ng/mL)	0.03 ± 0.01	0.57 ± 0.28	< 0.001
TNF-α (pg/mL)	4.07 (3.18–4.99)	1349.99 (661.60-2005.86)	< 0.001^*^
IL-10 (pg/mL)	1.33 ± 0.52	35.73 ± 18.09	< 0.001
IL-6 (pg/mL)	0.61 ± 0.27	41.57 ± 16.43	< 0.001
Etiology (*n*)	/		
Biliary		53	
Alcohol		30	
Hyperlipidemia		28	
Idiopathic		23	
Others		14	
APACHE II score	/	6.52 ± 2.98	
Modified Marshall score	/	1.15 ± 1.57	
MRSI	/	4.70 ± 2.73	
Severity	/		
Mild		41	
Moderate		72	
Severe		35	
Poor prognosis	/	29	
Secondary infection		6	
Organ failure		21	
Died		9	

The data distribution did not follow a normal distribution was presented as median (IQR) and analyzed by Mann-Whitney U test

*BMI* body mass index, *TC *total cholesterol, *TG *triglyceride, *WBC *white blood cell, *HCT *hematocrit, *LIPA *serum lipase, *AMY *serum amylase, *CRP *C-reaction protein, *PCT *procalcitonin, *TNF-α *tumor necrosis factor-α, *IL-10 *interleukin-10, *IL-6 *interleukin 6, *APACHE II *acute physiology and chronic health evaluation II, *MRSI *magnetic resonance severity index, / not applicable

### SNHG14 expression and its diagnostic potential

**Fig. 1:**
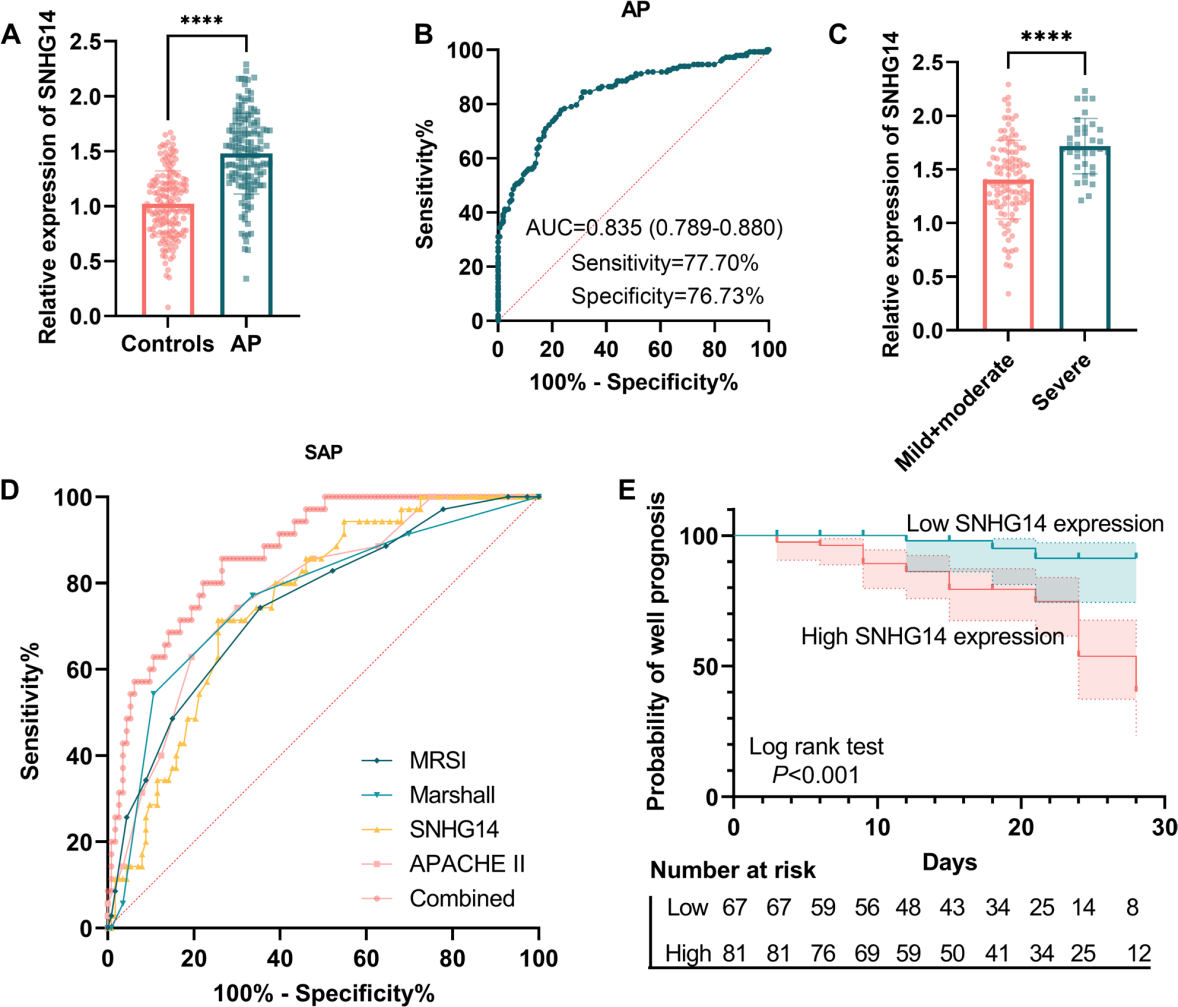
Analysis of SNHG14 expression and its diagnostic and prognostic value for AP. Data are presented as mean ± SD with individual data points overlaid. **A** Expression difference of SNHG14 in healthy controls (*n* = 159) and AP patients (*n* = 148). **B** ROC curve of SNHG14 for diagnosing AP (*n* = 148) from controls (*n* = 159). **C** SNHG14 levels in severe (*n* = 35) and mild + moderate (*n* = 113) AP patients. **D** Early diagnostic efficacy of SNHG14 for severe (vs. mild + moderate) and comparative and combined analysis with other indicators (APACHE II score, modified Marshall score, MRSI). **E** Kaplan-Meier curve showing time-to-first adverse event (infection, organ failure, or death) within 28 days. Median time-to-event: 5 days (IQR 3–8). ****, *P* < 0.0001

To evaluate the clinical relevance of SNHG14, its expression was first quantified in serum samples from 148 AP patients and 159 healthy controls. The serum SNHG14 level of AP cases was obviously upregulated compared to that in controls (Fig. 1A, mean FC = 1.48, *P* < 0.001). The potential diagnostic performance of SNHG14 was assessed by the ROC curve. The AUC of SNHG14 for distinguishing AP patients from healthy controls was 0.835 (95% CI: 0.790–0.880), with a sensitivity of 77.70%, a specificity of 76.73%, and the threshold was 1.225 (Fig. 1B).

Due to the highest SNHG14 level being observed in severe cases (Fig. 1C, *P* < 0.001), early diagnostic efficacy of SNHG14 for SAP was assessed. Efficacy of SNHG14 achieved an AUC of 0.757 (95% CI: 0.675–0.839), with an optimal cutoff of 1.615 (sensitivity 71.43%, specificity 74.34%), suggesting promising but preliminary diagnostic performance. Its diagnostic efficacy for AP diagnosis was not significantly different from the AUCs of commonly used clinical scoring systems such as the APACHE II score (AUC = 0.776, 95% CI: 0.692–0.860), modified Marshall score (AUC = 0.767, 95% CI: 0.673–0.861), and MRSI (AUC = 0.750, 95% CI: 0.658–0.842) (Fig. 1D; Table 2), as determined by DeLong’s test (Supplementary Table 2, *P* ≥ 0.743). A combined diagnostic model incorporating SNHG14, acute physiology and chronic health evaluation II (APACHE II), modified Marshall score, and magnetic resonance severity index (MRSI), constructed through multivariate logistic regression analysis (Supplementary Table 3), significantly improved the diagnostic efficacy (AUC = 0.872, 95% CI: 0.812–0.932, DeLong’s test *P* = 0.005) with a sensitivity of 85.71% and specificity of 73.45% (Table 2).

**Table 2:** Dignostic value of SAP diagnostic probability regression model

	Threshold	Sensitivity%	Specificity%	Youden Index	AUC (95%CI)
APACHE II	> 7.500	74.29	69.91	44.20	0.776 (0.692–0.860)
Marshall	> 2.500	54.29	89.38	43.67	0.767 (0.673–0.861)
MRSI	> 5.500	74.29	64.60	38.89	0.750 (0.658–0.842)
SNHG14	> 1.615	71.43	74.34	45.77	0.757 (0.675–0.839)
Combined	> 0.1947	85.71	73.45	59.16	0.872 (0.812–0.932)

### Relationship between SNHG14 and AP prognosis

According to the mean SNHG14 level, AP patients were categorized into low (*n* = 67) and high (*n* = 81) groups. Patients with high SNHG14 levels presented elevated TG (*P* = 0.003), WBC (*P* = 0.015), LIPA (*P* < 0.001), AMY (*P* < 0.001), CRP (*P* < 0.001), PCT (*P* < 0.001), TNF-α (*P* < 0.001), IL-10 (*P* < 0.001), and IL-6 (*P* < 0.001) levels compared with those with low levels (Table 3). The scores of APACHE II (*P* < 0.001), Marshall (*P* < 0.001), and MRSI (*P* = 0.024) scores were also significantly higher in the high expression group. Notably, elevated SNHG14 expression correlated with disease severity, as the high-expression group had a greater percentage of SAP patients (*P* < 0.001). Furthermore, adverse outcomes such as organ failure (*P* < 0.001) and mortality (*P* = 0.034) occurred more frequently in the high-expression group.

**Table 3:** Distribution of clinical features in acute pancreatitis patients with different SNHG14 levels

Features	SNHG14 expression		*P*
	Low (*n* = 67)	High (*n* = 81)	
Age	49.57 ± 11.90	49.49 ± 12.01	0.970
Gender (male/female)	38/29	45/36	0.887
BMI (kg/m^2^)	22.80 ± 2.60	22.95 ± 2.46	0.723
TC (mmol/L)	4.47 ± 1.44	4.08 ± 1.15	0.069
TG (mmol/L)	1.57 (0.63–2.62)	2.36 (1.58–3.14)	0.003
WBC (×10^9^/L)	11.26 ± 3.02	12.44 ± 2.81	0.015
HCT (%)	41.99 ± 2.32	41.58 ± 1.97	0.240
LIPA (U/L)	259.33 ± 98.71	327.77 ± 111.23	< 0.001
AMY (U/L)	615.35 (274.85-1279.81)	1340.99 (884.15-1978.25)	< 0.001
CRP (ng/mL)	129.62 ± 58.10	167.00 ± 62.81	< 0.001
PCT (ng/mL)	0.43 ± 0.26	0.68 ± 0.24	< 0.001
TNF-α (pg/mL)	1056.61 (578.11-1538.09)	1716.46 (827.93-2273.83)	< 0.001
IL-10 (pg/mL)	28.02 ± 15.24	42.11 ± 17.84	< 0.001
IL-6 (pg/mL)	34.40 ± 15.16	47.49 ± 15.09	< 0.001
Etiology (*n*)			0.251
Biliary	25	28	
Alcohol	15	15	
Hyperlipidemia	13	15	
Idiopathic	6	17	
Others	8	6	
APACHE II score	0.86 ± 0.23	0.52 ± 0.20	< 0.001
Modified Marshall score	1.00 (0.00–2.00)	2.00 (1.00–2.00)	< 0.001
MRSI	3.00 (2.00-5.50)	6.00 (5.00–8.00)	0.024
Severity			< 0.001
Mild	30	11	
Moderate	31	41	
Severe	6	29	
Poor prognosis	3	26	< 0.001
Secondary infection	1	5	0.151
Organ failure	2	19	< 0.001
Died	1	8	0.034

The data distribution did not follow a normal distribution was presented as median (IQR) and analyzed by Mann-Whitney U test

*BMI *body mass index, *TC *total cholesterol, *TG *triglyceride, *WBC *white blood cell, *HCT *hematocrit, *LIPA *serum lipase, *AMY *serum amylase, *CRP *C-reaction protein, *PCT *procalcitonin, *TNF-α *tumor necrosis factor-α, *IL-10 *interleukin-10, *IL-6 *interleukin 6, *APACHE II *acute physiology and chronic health evaluation II, *MRSI *magnetic resonance severity index

To identify independent predictors of poor AP prognosis, Cox regression analysis was performed (Table 4). Univariate analysis revealed that WBC (*P* = 0.001), AMY (*P* = 0.014), CRP (*P* = 0.031), PCT (*P* = 0.009), TNF-α (*P* = 0.007), IL-10 (*P* = 0.004), IL-6 (*P* = 0.004), SNHG14 (*P* = 0.002), APACHE II score (*P* = 0.007), modified Marshall score (*P* = 0.004), MRSI (*P* = 0.007), and disease severity (*P* = 0.003) were all potential predictors of poor prognosis. Variables with *P* < 0.05 in univariate analyses entered the initial multivariate model. After LASSO screening (Supplementary Fig. 1), WBC, IL-10, and SNHG14 were retained for the final multivariate Cox analysis. The results showed that WBC (HR = 4.188, 95% CI: 1.507–11.641, *P* = 0.006, VIF = 1.037), IL-10 (HR = 2.991, 95% CI: 1.100-8.132, *P* = 0.032, VIF = 1.098), and SNHG14 (HR = 4.794, 95% CI: 1.493–15.393, *P* = 0.008, VIF = 1.134) were independent predictors of 28-day prognosis in AP patients (Table 4). The event-per-variable (EPV) ratio of this final model was 9.7 (29 events/3 variables). Moreover, the VIF of all variables was < 5.0, indicating negligible multicollinearity issues. Survival curve analysis indicated that patients with elevated SNHG14 expression had a significantly higher cumulative incidence of unfavorable outcomes compared to those with lower levels (Fig. 1E, Log-rank *P* < 0.001). This finding confirms the strong association between increased SNHG14 expression and poor prognosis.

**Table 4:** Univariate and multivariate Cox regression analyses of factors associated with 28-day poor prognosis in AP patients (after LASSO variable selection)

Features	Univariate analysis		Multivariate analysis	
	*P*	HR (95% CI)	*P*	HR (95% CI)
Age	0.358	1.015 (0.983–1.047)		
Sex	0.600	0.819 (0.390–1.723)		
BMI	0.811	1.093 (0.527–2.269)		
TC	0.850	1.074 (0.511–2.260)		
TG	0.445	1.331 (0.640–2.768)		
WBC*	0.001	4.430 (1.803–10.885)	0.006	4.188 (1.507–11.641)
HCT	0.426	0.744 (0.359–1.542)		
LIPA	0.400	1.374 (0.655–2.882)		
AMY	0.014	2.904 (1.237–6.817)		
CRP	0.031	2.455 (1.085–5.551)		
PCT	0.009	3.106 (1.326–7.278)		
TNFα	0.007	3.814 (1.450-10.032)		
IL10*	0.004	3.565 (1.513–8.401)	0.032	2.991 (1.100-8.132)
IL6	0.004	3.761 (1.528–9.259)		
SNHG14*	0.002	6.357 (1.922–21.031)	0.008	4.794 (1.493–15.393)
APACHE II	0.007	4.228 (1.471–12.155)		
Marshall	0.004	3.195 (1.454–7.022)		
MRSI	0.007	3.266 (1.390–7.674)		
Severity	0.003	3.031 (1.462–6.282)		

*HR* hazard ratio, *CI *confidence interval

*, Variables selected by Least Absolute Shrinkage and Selection Operator (LASSO) regression for inclusion in the final multivariate model

### Influence of SNHG14 on cell function and inflammatory factors in cell models

**Fig. 2:**
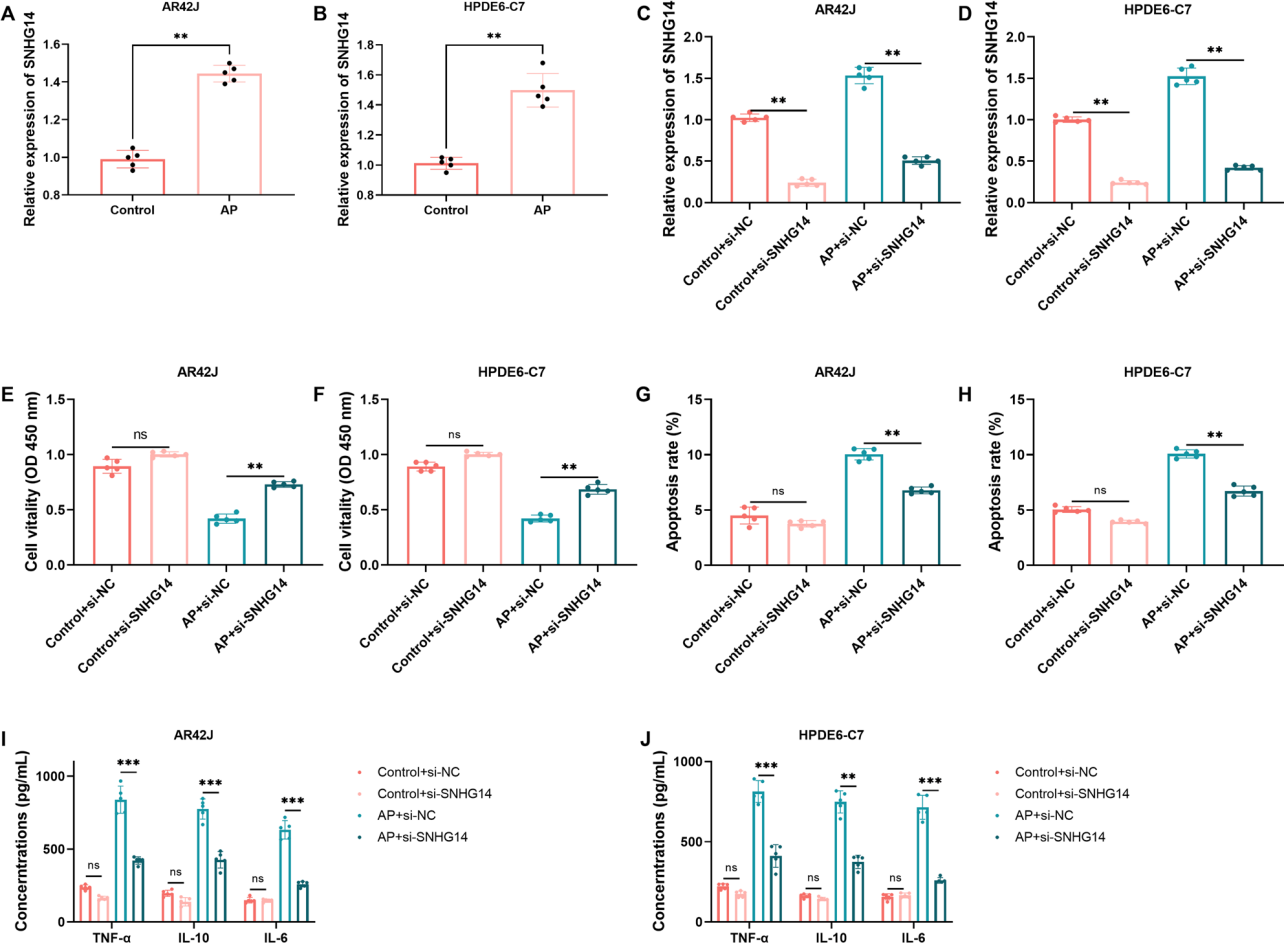
Effects of SNHG14 on cell proliferation, apoptosis, and inflammatory factors (*n* = 5). **A** Expression of SNHG14 in AR42J cell model. **B** Expression of SNHG14 in HPDE6-C7 cell model. **C** Validation of SNHG14 expression in si-SNHG14 stably expressed AR42J cells. **D** Validation of SNHG14 expression in si-SNHG14 stably expressed HPDE6-C7 cells. **E** Impact of si-SNHG14 on cell viability of AR42J cells. **F** Impact of si-SNHG14 on cell viability of HPDE6-C7 cells. **G** Impact of si-SNHG14 on cell apoptosis of AR42J cells. **H** Impact of si-SNHG14 on cell apoptosis of HPDE6-C7 cells. **I** Impact of si-SNHG14 on inflammatory factors in AR42J cells. **J** Impact of si-SNHG14 on inflammatory factors in HPDE6-C7 cells. ns, not significant; **, *P* < 0.01; ***, *P* < 0.001

To functionally validate the role of SNHG14, Cerulein was used to treat AR42J and HPDE6-C7 cells, successfully establishing AP cell models. Cerulein treatment significantly induced SNHG14 expression in both cell models (Fig. 2A-B, *P* = 0.008).

For investigating the role of SNHG14 in AP, cell models with stable SNHG14 depletion were constructed using AR42J and HPDE6-C7 cell lines. QRT-PCR assays confirmed that the SNHG14 expression was significantly reduced by about 76% (Fig. 2C-D, *P* < 0.05). These results confirmed the successful establishment of the knockdown model. Subsequent functional assays were then implemented. CCK-8 assay showed that silence of SNHG14 significantly enhanced cell viability in the Cerulein-induced AP cell models (Fig. 2E-F, *P* < 0.05). Flow cytometry analysis revealed that silence of SNHG14 significantly attenuated Cerulein-induced cell apoptosis (Fig. 2G-H, *P* < 0.05). Furthermore, the ELISA assay indicated that SNHG14 knockdown notably decreased the secretion levels of TNF-α, IL-10, and IL-6 in cell supernatant after Cerulein stimulation (Fig. 2I-J, *P* < 0.05). These findings suggest that SNHG14 knockdown alleviates Cerulein-induced cell damage and inhibits the inflammatory response.

### SNHG14 as a molecular sponge for adsorbing miR-30a-5p

**Fig. 3:**
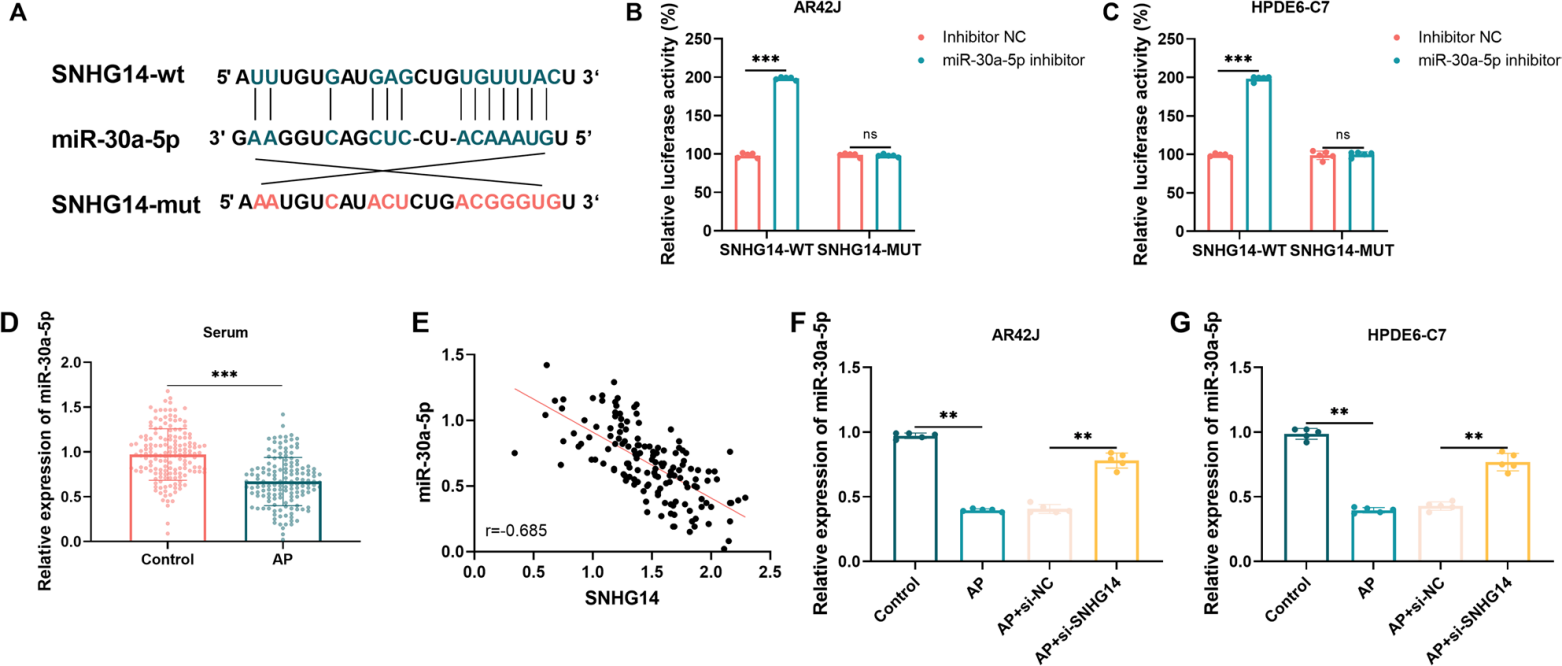
Target association between miR-30a-5p and SNHG14. **A** Target binding sequence. **B** Dual-luciferase assay in AR42J cells (*n* = 5). **C** Dual-luciferase assay in HPDE6-C7 cells (*n* = 5). **D** Serum miR-30a-5p expression (*n* = 159 for control, *n* = 148 for AP patients). **E** Correlation analysis between SNHG14 and miR-30a-5p. **F** Regulatory effect of SNHG14 expression on miR-30a-5p in AR42J cells (*n* = 5). **G** Regulatory effect of SNHG14 expression on miR-30a-5p in HPDE6-C7 cells (*n* = 5). ns, not significant; **, *P* < 0.01; ***, *P* < 0.001

To explore the molecular mechanism of SNHG14, potential target miRNAs of SNHG14 were predicted using starBase. The starBase database revealed a possible binding site for miR-30a-5p (Fig. 3A). The dual-luciferase reporter assay was performed to certify the target association. The results demonstrated that, in contrast to the inhibitor NC group, the cotransfection of the SNHG14-wt reporter gene alongside the miR-30a-5p inhibitor markedly increased luciferase activity. In contrast, the SNHG14-mut reporter gene did not produce a similar enhancement (Fig. 3B-C, *P* < 0.001), demonstrating that SNHG14 can directly bind to miR-30a-5p.

Clinical samples and cell models confirmed the target association between SNHG14 and miR-30a-5p. AP patients exhibited significantly reduced serum levels of miR-30a-5p relative to healthy controls (Fig. 3D, mean FC = 0.74, *P* < 0.001). SNHG14 and miR-30a-5p expression were inversely correlated in AP patient serum (Fig. 3E, *r*=-0.685, *P* < 0.001). SNHG14 knockdown in cellular models consistently increased miR-30a-5p expression, confirming their regulatory relationship (Fig. 3F-G, *P* < 0.01). It suggests that SNHG14 may function by adsorbing miR-30a-5p and negatively regulating its expression.

### SNHG14 influences the biological behavior of AP cells via miR-30a-5p

**Fig. 4:**
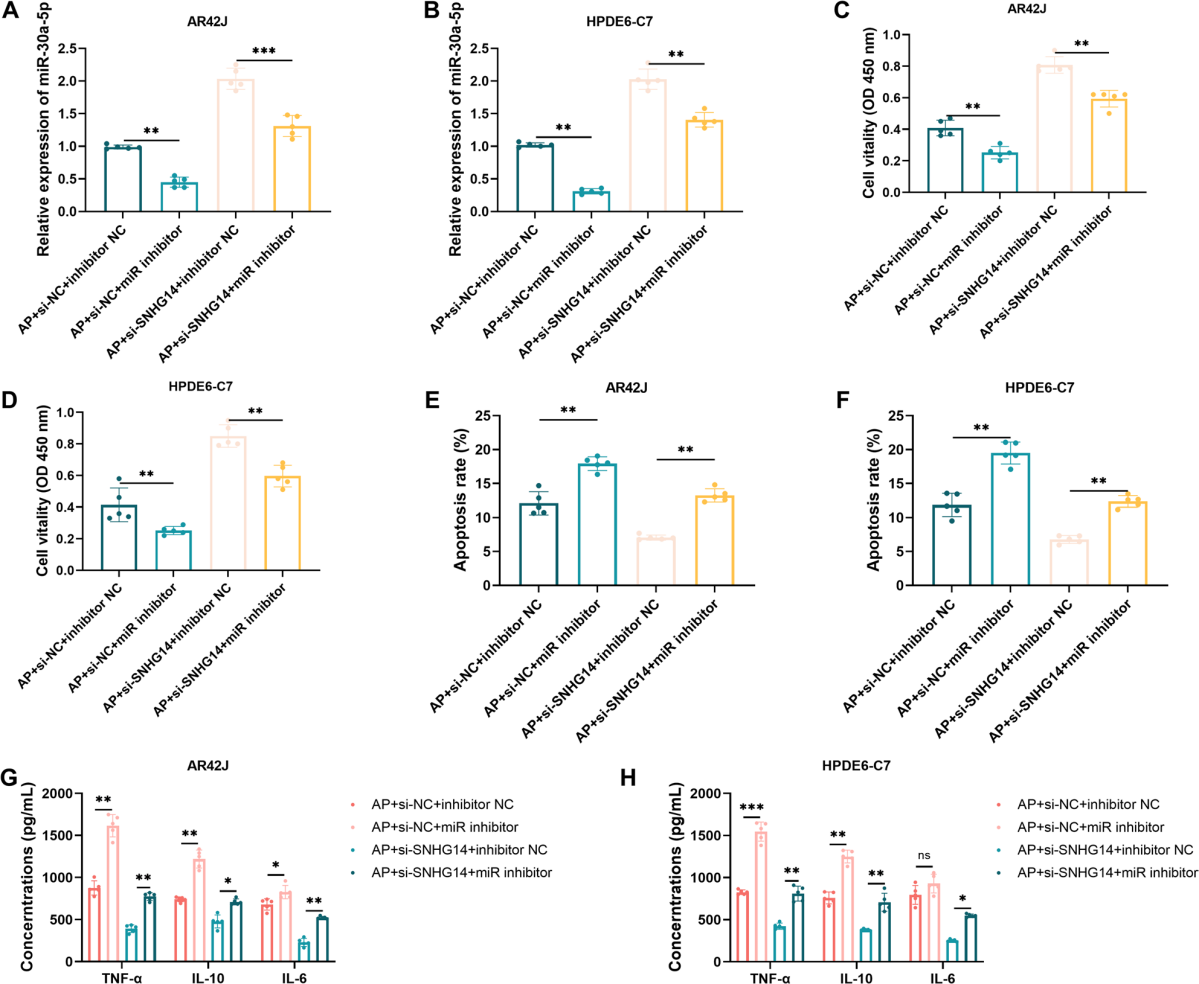
SNHG14 regulates pancreatitis cells via miR-30a-5p (*n* = 5). **A** Verification of miR-30a-5p expression in co-transfected AR42J cells. **B** Verification of miR-30a-5p expression in co-transfected HPDE6-C7 cells. **C** Impact of miR-30a-5p on cell viability of AR42J cells. **D** Impact of miR-30a-5p on cell viability of HPDE6-C7 cells. **E** Impact of miR-30a-5p on cell apoptosis of AR42J cells. **F** Impact of miR-30a-5p on cell apoptosis of HPDE6-C7 cells. **G** Impact of miR-30a-5p on inflammatory factors in AR42J cells. **H** Impact of miR-30a-5p on inflammatory factors in HPDE6-C7 cells. *, *P* < 0.05; **, *P* < 0.01; ***, *P* < 0.001

Rescue experiments were performed to verify whether SNHG14 exerts its cellular effects via miR-30a-5p. Results showed that miR-30a-5p levels were inhibited when cells were cotransfected with si-SNHG14 and miR-30a-5p inhibitor. Inhibition of miR-30a-5p reversed the elevated miR-30a-5p expression induced by si-SNHG14 (Fig. 4A-B, *P* < 0.01).

Rescue experiments demonstrated that miR-30a-5p inhibition abolished the pro-survival (Fig. 4C-D, *P* < 0.01) and anti-apoptotic (Fig. 4E-F, *P* < 0.01) effects of SNHG14 knockdown. Similarly, miR-30a-5p inhibition reversed the suppression of inflammatory cytokine secretion (TNF-α, IL-10, IL-6) caused by SNHG14 silencing (Fig. 4G-H, *P* < 0.05). These findings demonstrate that SNHG14 at least partially exacerbates damage in the AP cell model by negatively regulating miR-30a-5p expression.

### Bioinformatics analysis of miR-30a-5p target genes

**Fig. 5:**
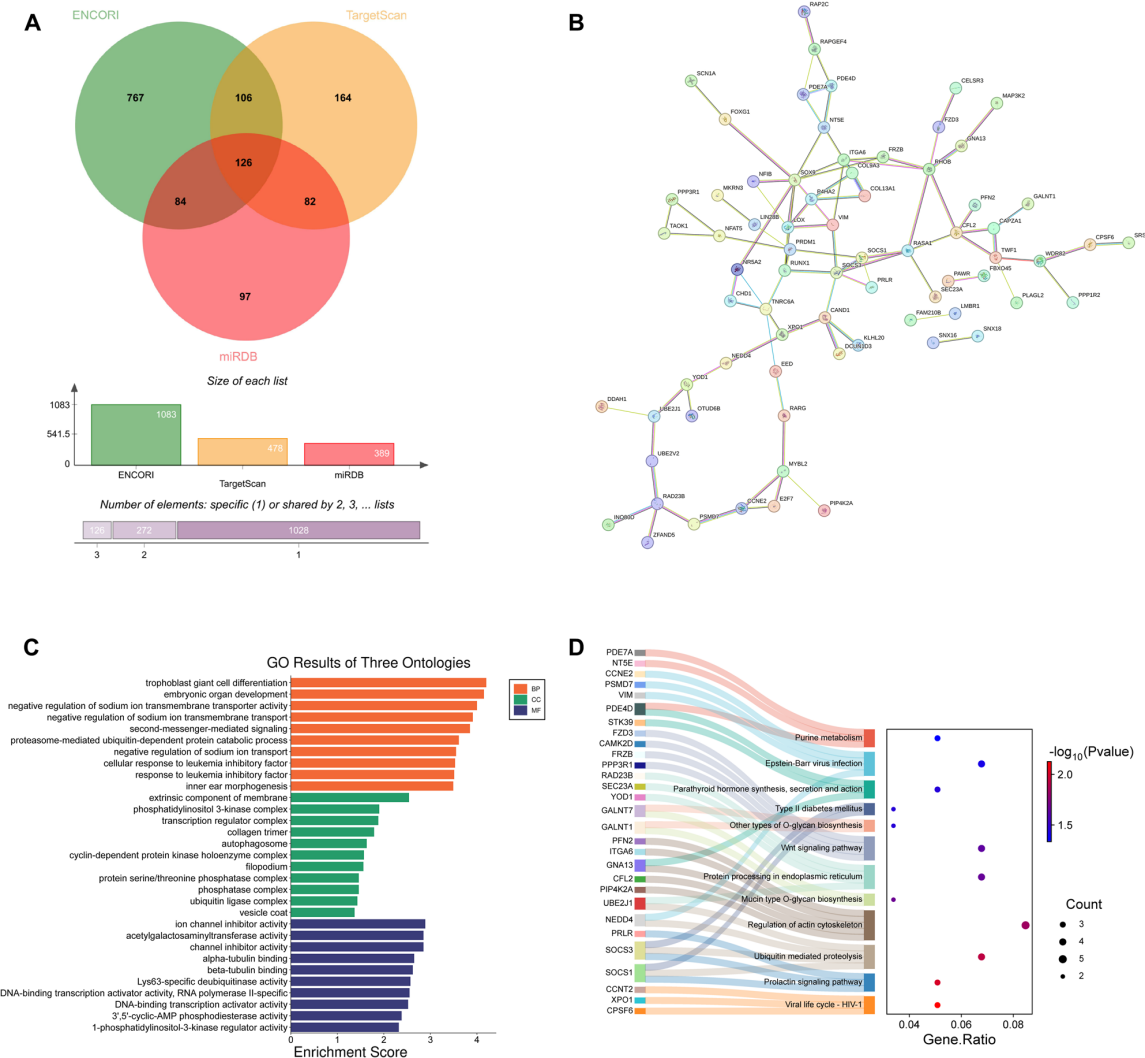
Bioinformatics analysis of miR-30a-5p target genes. **A** Venn diagram shows the overlap of miR-30a-5p target genes screened from different databases. **B** PPI network illustrates the interactions among miR-30a-5p target genes. **C** GO analysis reveals the biological functions of miR-30a-5p target genes. **D** KEGG pathway analysis explores the signaling pathways involved in miR-30a-5p target genes

Systematic bioinformatic analysis of miR-30a-5p target genes was performed, to identify the potential downstream network regulated by miR-30a-5p and clarify the underlying pathway of the SNHG14/miR-30a-5p axis in AP. A total of 126 high-confidence candidate target genes of miR-30a-5p were obtained from ENCORI, TargetScan, and miRDB databases using a Venn diagram (Fig. 5A). PPI network analysis of candidate genes revealed complex interactions among them. Nine hub genes (SOX9, ITGA6, RHOB, SOCS3, CFL2, LOX, RASA1, RUNX1, and TNRC6A) were identified by PPI network with the node degree ≥ 5 (Fig. 5B). Systematic bioinformatics analysis was performed on the 126 overlapping target genes identified through database screening to elucidate the potential downstream regulatory mechanisms of miR-30a-5p in AP. GO analysis (Fig. 5C) indicated a significant enrichment of the target genes in processes including proteasome-mediated ubiquitin-dependent protein catabolism and cellular responses to leukemia inhibitory factor. For cellular components, these genes were enriched in autophagosomes and ubiquitin ligase complexes. For molecular functions, the genes were primarily involved in ion channel inhibitor activity. KEGG pathway analysis revealed enrichment in several key pathways, such as axon guidance (enrichment score = 2.24), ubiquitin-mediated proteolysis (enrichment score = 1.92), protein processing within the endoplasmic reticulum (enrichment score = 1.65), Wnt signaling pathway (enrichment score = 1.63), and actin cytoskeleton regulation (enrichment score = 1.83) (Fig. 5D). These findings suggest a potential mechanism by which the SNHG14/miR-30a-5p axis regulates inflammation, autophagy, and cytoskeletal rearrangement in AP.

## Discussion

This study provides the first evidence that serum SNHG14 was significantly upregulated in AP patients. More importantly, SNHG14 showed potential for distinguishing AP and early identification of SAP, so it could be explored as a circulating biomarker. The observed diagnostic performance of SNHG14 for SAP is comparable to conventional clinical scoring systems such as MRSI, APACHE II, and modified Marshall scores, which have been widely utilized in predicting AP progression [30, 31]. This suggests that SNHG14 may act as an important liquid biopsy biomarker for SAP, providing a beneficial addition to the current diagnostic paradigm. When combined with clinical scores, the diagnostic efficacy of SNHG14 increases to 0.872, demonstrating the potential of their combined application. However, this single-center study has a moderate AUC value and no external validation. The utility of SNHG14 as a diagnostic marker requires further study.

AP patients with high SNHG14 levels exhibited a more severe clinical phenotype. Multivariate Cox regression analysis revealed that WBC count, IL-10, and SNHG14 were independent predictors for poor 28-day prognosis in AP patients. This finding was supported by KM survival curves. Xu et al. found that IL-10 was upregulated in AP cases compared to controls [32]. While Bava et al. suggested that IL-10 participates in the mechanism of pirfenidone for AP therapy [33]. These findings imply that elevated levels may represent a complex state of immune dysregulation in AP.

Cell models confirmed that elevated SNHG14 levels worsened cerulein-induced cell injury, apoptosis, and inflammatory factor secretion. Dual-luciferase reporter gene assays and rescue experiments further confirmed that this function is mediated by direct binding and suppression of miR-30a-5p. SNHG14 is upregulated in acute kidney injury [22] and spinal cord injury [19]. Inhibiting SNHG14 significantly increases cell viability and inhibits apoptosis in LPS-treated HK-2 and PC-12 cells. This consistent pattern across different tissues and diseases suggests that SNHG14 may act as a broad regulator of cellular damage, with highly context-dependent functions. However, Deng and colleagues found that silencing SNHG14 suppressed proliferation and facilitated the apoptosis of pancreatic cancer cells via miR613/ANXA2 axis [34]. This difference likely stems from the inherent nature of the diseases. AP is an acute inflammatory disease centered on cellular stress, inflammation amplification, and the balance between apoptosis and necrosis; while pancreatic cancer involves proliferation, invasion, and escape from apoptosis. Besides, the role of miR-30a-5p in AP aligns with previous reports linking miR-30a-5p to autophagy and inflammation control [35–38]. This demonstrates the complexity and context-dependent nature of SNHG14 functions. Present cerulein-induced in vitro models replicate the key cellular abnormalities associated with AP, such as inflammation and cell death, but do not emulate the complex inflammatory response seen in human AP. The results should be verified using in vivo and clinical experiments.

More importantly, this study offers a testable hypothesis for the downstream action network of the SNHG14/miR-30a-5p axis through comprehensive bioinformatics analyses. PPI analysis found 9 hub target genes of miR-30a-5p. SOCS3 acts as a critical negative regulator in the JAK/STAT inflammatory signaling pathway [39], which regulates the TNF-α and IL-6 secretion. RHOB is involved in cytoskeleton rearrangement and stress responses during apoptosis [40]. Dysregulation of these hub genes may directly affect inflammation in AP. These genes are located at the intersection of AP pathological processes, such as autophagy and protein homeostasis. GO enrichment analysis revealed significant enrichment of the target genes in biological processes such as proteasome-mediated, ubiquitin-dependent protein breakdown, and cellular response to leukemia inhibitory factor, suggesting their involvement in protein metabolism and inflammatory regulation [41]. Candidate genes were abundant in cellular components relevant to impaired autophagy and abnormal protein degradation in pancreatitis [42, 43]. For molecular function, the candidate genes were primarily related to ion channel inhibitor activity, potentially influencing cell membrane stability and inflammatory signaling during AP [44]. KEGG pathway analysis showed significant enrichment in ubiquitin-mediated proteolysis, protein processing in the endoplasmic reticulum, the Wnt pathway, and regulation of the actin cytoskeleton. These pathways are broadly implicated in cellular stress, inflammation, apoptosis, and autophagy, closely aligning with the pathogenesis of AP. Based on these findings, a detailed working model is proposed. Through competitive endogenous RNA (ceRNA) activity, SNHG14 activates a pathway that regulates miR-30a-5p activity and can cause cell death in AP. reduces miR-30a-5p activity, which may derepress target genes and disrupt ubiquitination-autophagy balance and endoplasmic reticulum stress adaptation. Disruption of these pathways drives inflammation and cell death in AP. This computationally predicted mechanism provides a clear direction for subsequent experimental validation. The pathways enriched among miR-30a-5p predicted targets are not isolated cellular events. They form interconnected modules commonly co-perturbed in complex diseases. This pattern indicates SNHG14 may govern a coordinated network of protein quality control and stress adaptation via miR-30a-5p modulation. Multi-omics studies show that disease progression is driven by interconnected modules, not single genes [45]. The SNHG14/miR-30a-5p axis probably works as part of a larger context-specific network that determines AP severity. Future studies are needed to map this axis onto the in vivo regulatory landscape and identify its key upstream inducers and downstream effectors.

The main strength of this study is the first to show the clinical and functional roles of SNHG14 in AP. Targeting the SNHG14/miR-30a-5p axis offers a potential strategy for AP. This enables ultrasensitive detection of SNHG14. Investigating its cross-talk with specific receptor pathways may further promote clinical translation. Computational prediction has created a clear and actionable mechanistic model downstream of the SNHG14/miR-30a-5p axis. Identification of the circulating SNHG14/miR-30a-5p axis confers multiple translational advantages. First, as a circulating RNA biomarker, it has the potential for noninvasive and dynamic monitoring, complementing static imaging and single-time-point assays. The potential of SNHG14 as a biomarker depends on sensitivity, specificity, and rapid quantification. Liquid biopsy claims need engineering solutions. Biosensor-driven analysis has shown promise, especially in oncology [18]. Second, adapting SNHG14-specific platforms, such as electrochemical biosensors, can guide future development. Our diagnostic cut-offs and prognostic associations guide their analytical performance. Antisense oligonucleotides or miRNA mimics targeting this axis modulate inflammatory and apoptotic pathways. Future integration with biosensing platforms, including green-synthesized nanomaterials [46], could enable point-of-care detection. Green-synthesized nanomaterials [47] offer biocompatibility, cost-effectiveness, and eco-friendly production for sensitive diagnostics of RNA biomarkers. This approach is not intended to replace current emergency and supportive treatments. It serves as a potential complementary strategy.

However, several limitations should be acknowledged to guide future research. Our data indicate a promising association between SNHG14 and AP presence or severity, the claim of its standalone diagnostic value must be tempered by key study limitations. Firstly, this clinical evaluation is based on a single-center cohort, lacking external validation, so its findings are limited. The reported AUC values are optimistic and subject to overfitting. SNHG14 may act as a candidate biomarker in further multi-center studies. Despite LASSO penalization and bootstrap validation being applied to mitigate overfitting risks of 28-day poor AP prognosis, the small event count (*n* = 29) remains an intrinsic limitation. Despite the acceptable final EPV of 9.7, this value constrains the range of covariates eligible for robust evaluation. Multi-center, larger-scale prospective cohorts are urgently needed for external validation. Secondly, this cross-sectional study relied on serum samples and presents a simplified mechanistic model (SNHG14/miR-30a-5p). In the adaptive biological system of AP, this axis likely operates as part of a dynamic network with feedback loops and redundancy. Our in vitro models cannot capture this complexity, highlighting the need for more sophisticated investigations in the local pancreatic microenvironment. Thirdly, mechanistic insights are limited by the in vitro setting. Whole-organism validation using AP animal models is crucial for confirmation. Fourthly, this study focused only on 28-day short-term prognosis, without tracking long-term outcomes such as recurrence rate, pancreatic exocrine insufficiency, or diabetes mellitus. Given the significant impact of AP on long-term quality of life, the relationship between SNHG14 and long-term outcomes warrants further investigation. Future studies with extended follow-up periods and comprehensive functional assessments are needed to clarify the long-term predictive value of SNHG14 in AP management. Fifthly, although batch processing with controls was performed, potential batch effects in ELISA and qPCR assays cannot be completely excluded. Lastly, although bioinformatic analysis predicted miR-30a-5p target genes and enriched pathways, and rescue experiments preliminarily confirmed functional dependency, direct validation of specific target genes and detailed mechanistic insights are still lacking. Interactions between SNHG14 and other miRNAs were not explored. Cross-platform validation using publicly available AP transcriptome datasets and development of a machine-learning classifier combining SNHG14 with clinical features are warranted to build more precise risk prediction tools. Additionally, the lack of serial sampling highlights the need for dynamic monitoring in future research. In summary, these limitations point to important directions for future research. Through multi-center collaboration, multi-dimensional validation, and in-depth mechanistic exploration, the SNHG14/miR-30a-5p axis holds substantial potential for clinical translation in AP.

## Conclusions

This research reveals that SNHG14 is upregulated in AP, with levels closely tied to disease severity and poor prognosis. SNHG14 shows significant diagnostic and predictive value. Mechanistically, SNHG14 may sponge miR-30a-5p to regulate downstream target genes, influencing inflammatory responses and apoptotic processes. These findings illuminate the molecular mechanisms of AP and provide experimental evidence for developing lncRNA-based diagnostic tools and therapies. However, it represents an auxiliary molecular therapy strategy rather than a replacement for existing management. Future studies should expand sample sizes to validate clinical applicability and further investigate the specific mechanisms and therapeutic potential of the SNHG14/miR-30a-5p axis in AP. 

## Supplementary Information

Supplementary Material 1: Figure S1. Least absolute shrinkage and selection operator (LASSO) regression analysis for variable selection in the Cox model. A, Cross-validation curve (λ=0.081). B, Coefficient path plot.

Supplementary Material 2.

Supplementary Material 3.

Supplementary Material 4.

## Data Availability

The datasets used and/or analysed during the current study are available from the corresponding author on reasonable request.

## Abbreviations

AP: Acute pancreatitis
SAP: Severe acute pancreatitis
lncRNAs: Long non-coding RNAs
SNHG14: Small nucleolar RNA host gene 14
miRNA / miR: MicroRNA
miR-30a-5p: Microrna-30a-5p
UBE3A: Ubiquitin protein ligase E3A gene
CT: Computed tomography
ELISA: Enzyme-linked immunosorbent assay
LIPA: Lipase
AMY: Amylase
HCT: Hematocrit
TC: Total cholesterol
TG: Triglycerides
CRP: C-reactive protein
PCT: Procalcitonin
TNF-α: Tumor necrosis factor-alpha
IL: Interleukin
qRT-PCR: Quantitative real-time polymerase chain reaction
FBS: Fetal bovine serum
PBS: Phosphate buffer solution
MOI: Multiplicity of infection
NC: Negative control
CCK-8: Cell counting kit-8
wt: Wild-type
mut: Mutant-type
PPI: Protein-protein interaction
FDR: False discovery rate
GO: Gene ontology
KEGG: Kyoto Encyclopedia of Genes and Genomes
FC: Fold change
ROC: Receiver operating characteristic curve
KM: Kaplan-Meier
AUC: Area under the curve
HR: Hazard ratio
95% CI: 95% confidence interval
LASSO: Least absolute shrinkage and selection operator
VIF: Variance inflation factor
IQR: Interquartile range
APACHE II: Acute physiology and chronic health evaluation II
MRSI: Magnetic resonance severity index
EPV: Event-per-variable
ceRNA: Competitive endogenous RNA

## References

[1] Liu Y, et al. Sirtuin4 alleviates severe acute pancreatitis by regulating HIF-1alpha/HO-1 mediated ferroptosis. Cell Death Dis. 2023;14(10):694. 37865653 10.1038/s41419-023-06216-x PMC10590376

[2] Wu S, et al. Postponed endoscopic necrosectomy results in a lower rate of additional intervention for infected walled-off necrosis. Sci Rep. 2024;14(1):11610. 38773218 10.1038/s41598-024-61675-2 PMC11109209

[3] Liu Y, et al. Thioredoxin-interacting protein deficiency protects against severe acute pancreatitis by suppressing apoptosis signal-regulating kinase 1. Cell Death Dis. 2022;13(10):914. 36316322 10.1038/s41419-022-05355-x PMC9622726

[4] Peng C, et al. MLKL signaling regulates macrophage polarization in acute pancreatitis through CXCL10. Cell Death Dis. 2023;14(2):155. 36828808 10.1038/s41419-023-05655-w PMC9958014

[5] Selin D, et al. Long-Term Mortality in Acute Pancreatitis-A Population-Based Cohort Study. United Eur Gastroenterol J. 2025;13(4):640–9. 10.1002/ueg2.12774 PMC12090827 40019214

[6] Mederos MA, Reber HA, Girgis MD. Acute Pancreatitis: Rev JAMA. 2021;325(4):382–90. 10.1001/jama.2020.20317 33496779

[7] Ko J, et al. Associations between Intra-Pancreatic Fat Deposition, Pancreas Size, and Pancreatic Enzymes in Health and after an Attack of Acute Pancreatitis. Obes Facts. 2022;15(1):70–82. 34753126 10.1159/000519621 PMC8820142

[8] Wang G, et al. Effects of QingYi decoction on inflammatory markers in patients with acute pancreatitis: A meta-analysis. Phytomedicine. 2022;95:153738. 34544631 10.1016/j.phymed.2021.153738

[9] Yang L, et al. Extracellular SQSTM1 exacerbates acute pancreatitis by activating autophagy-dependent ferroptosis. Autophagy. 2023;19(6):1733–44. 36426912 10.1080/15548627.2022.2152209 PMC10262765

[10] Xia CC, et al. Reactive oxygen species and oxidative stress in acute pancreatitis: Pathogenesis and new therapeutic interventions. World J Gastroenterol. 2024;30(45):4771–80. 39649547 10.3748/wjg.v30.i45.4771 PMC11606378

[11] He J, et al. Hspb1 protects against severe acute pancreatitis by attenuating apoptosis and ferroptosis via interacting with Anxa2 to restore the antioxidative activity of Prdx1. Int J Biol Sci. 2024;20(5):1707–28. 38481805 10.7150/ijbs.84494 PMC10929186

[12] Khataniar H, Vellankal S. Acute Hemorrhagic Pancreatitis as a Rare Complication of Dengue Fever. ACG Case Rep J. 2023;10(9):e01152. 37753103 10.14309/crj.0000000000001152 PMC10519561

[13] Zhu J, et al. Acute Lymphoblastic Leukemia in Combined Methylmalonic Acidemia and Homocysteinemia (cblC Type): A Case Report and Literature Review. Front Genet. 2022;13:856552. 35495149 10.3389/fgene.2022.856552 PMC9048794

[14] Javan M, et al. Acetylation and Sirtuins: Molecular Mechanisms Driving Metabolic Flexibility. Adv Biology Earth Sci. 2025;10(3):547–84.

[15] Alan F, ÇOlak AM. Phytochemical and Antioxidant Characterization of Myrtle (Myrtus communis L.) Fruit. ISPEC J Agricultural Sci. 2025;9(4):1182–93.

[16] TekİN SB, PoyrazoĞLu ES, GÜÇEr Y. Investigation of the Functional Properties of Some Origanum Species and Their Bioactive Components. ISPEC J Agricultural Sci. 2025;9(2):630–43.

[17] Pashayev A, et al. Biological insights from Saliva Diagnostics to assess Human Factors and flight safety performance in Aviation Simulators. Advances in Biology & Earth Science. 2025;10(2):185–9.

[18] Baran A, et al. Green-synthesizednanoparticles for biomedical sensor technology. Nanosensors Healthc Diagnostics. 2025(13):355–80.

[19] Jiang H, et al. Knockdown of lncRNA SNHG14 alleviates LPS-induced inflammation and apoptosis of PC12 cells by regulating miR-181b-5p. Exp Ther Med. 2021;21(5):497. 33791006 10.3892/etm.2021.9928 PMC8005701

[20] Abd El-Aziz A, et al. Influence of pentoxifylline on gene expression of PAG1/ miR-1206/ SNHG14 in ischemic heart disease. Biochem Biophys Rep. 2021;25:100911. 33553684 10.1016/j.bbrep.2021.100911 PMC7846894

[21] Zhang X, et al. SNHG14 enhances gemcitabine resistance by sponging miR-101 to stimulate cell autophagy in pancreatic cancer. Biochem Biophys Res Commun. 2019;510(4):508–14. 30737032 10.1016/j.bbrc.2019.01.109

[22] Yang N, et al. FTO attenuates LPS-induced acute kidney injury by inhibiting autophagy via regulating SNHG14/miR-373-3p/ATG7 axis. Int Immunopharmacol. 2024;128:111483. 38215656 10.1016/j.intimp.2023.111483

[23] Hu F, et al. LncRNA-PVT1 aggravates severe acute pancreatitis by promoting autophagy via the miR-30a-5p/Beclin-1 axis. Am J Transl Res. 2020;12(9):5551–62. 33042437 PMC7540137

[24] Sun L, et al. LncRNA MIAT suppresses inflammation in LPS-induced J774A.1 macrophages by promoting autophagy through miR-30a-5p/SOCS1 axi. Sci Rep. 2024;14(1):22608. 39349964 10.1038/s41598-024-73607-1 PMC11442610

[25] Xu F, Bian N, Li X. SNHG14 Elevates NFAT5 Expression Through Sequestering miR-375-3p to Promote MPP + -Induced Neuronal Apoptosis, Inflammation, and Oxidative Stress in Parkinson’s Disease. Neurochem Res. 2024;49(5):1212–25. 38381247 10.1007/s11064-024-04106-y

[26] Cuiwen Kong, Liping Lysun, Fenfen Yu. The effect of LncRNA SNHG14 on high glucose induced podocyte injury by targeting miR-30a-5p. Tianjin Med J. 2025. 53(9):903–909.

[27] Banks PA, et al. Classification of acute pancreatitis–2012: revision of the Atlanta classification and definitions by international consensus. Gut. 2013;62(1):102–11. 23100216 10.1136/gutjnl-2012-302779

[28] Song TJ, et al. Effect of SNHG11/miR-7-5p/PLCB1 Axis on Acute Pancreatitis through Inhibiting p38MAPK Pathway. Cells. 2022;12(1):65. 10.3390/cells12010065 PMC9818913 36611865

[29] Tang D, et al. SRplot: A free online platform for data visualization and graphing. PLoS ONE. 2023;18(11):e0294236. 37943830 10.1371/journal.pone.0294236 PMC10635526

[30] Londono-Ruiz G, et al. Prediction of severe pancreatitis in a population with low atmospheric oxygen pressure. Sci Rep. 2022;12(1):19518. 36376428 10.1038/s41598-022-21789-x PMC9663689

[31] Gu K, Shang W, Wang D. Visceral obesity anthropometric indicators as predictors of acute pancreatitis severity. Front Med (Lausanne). 2025;12:1536090. 40718412 10.3389/fmed.2025.1536090 PMC12289579

[32] Xu R, et al. Soluble B7-H5 Is a Novel Diagnostic, Severity, and Prognosis Marker in Acute Pancreatitis. Biomed Res Int. 2021;2021:p1223850. 10.1155/2021/1223850 PMC8519671 34660778

[33] Palathingal Bava E et al. Pirfenidone increases IL-10 and improves acute pancreatitis in multiple clinically relevant murine models. JCI Insight. 2022;7(2). 10.1172/jci.insight.141108 PMC8855813 34847076

[34] Deng PC, et al. LncRNA SNHG14 potentiates pancreatic cancer progression via modulation of annexin A2 expression by acting as a competing endogenous RNA for miR-613. J Cell Mol Med. 2019;23(11):7222–32. 31513352 10.1111/jcmm.14467 PMC6815841

[35] Li X, et al. Molecular mechanism analysis of m6A modification-related lncRNA-miRNA-mRNA network in regulating autophagy in acute pancreatitis. Islets. 2022;14(1):184–99. 36218109 10.1080/19382014.2022.2132099 PMC9559333

[36] Fan L, et al. Identification of Acute Pancreatitis-Related Genes and Pathways by Integrated Bioinformatics Analysis. Dig Dis Sci. 2020;65(6):1720–32. 31724100 10.1007/s10620-019-05928-5

[37] Fang Y, Zou L, He W. miR–30a–5p mitigates autophagy by regulating the Beclin–1/ATG16 pathway in renal ischemia/reperfusion injury. Int J Mol Med. 2021;48(1). 10.3892/ijmm.2021.4977 PMC8175063 34080645

[38] Liu X, et al. LncRNA MALAT1 regulates cigarette smoke induced airway inflammation by modulating miR-30a-5p/JNK signaling pathway. Int Immunopharmacol. 2024;140:112826. 39128416 10.1016/j.intimp.2024.112826

[39] Yan D, et al. SOCS3 inhibiting JAK-STAT pathway enhances oncolytic adenovirus efficacy by potentiating viral replication and T-cell activation. Cancer Gene Ther. 2024;31(3):397–409. 38102464 10.1038/s41417-023-00710-2

[40] Liu P, et al. RHOB regulates megakaryocytic and erythroid differentiation by altering the cell cycle and cytoskeleton. Sci Rep. 2025;15(1):13159. 40240762 10.1038/s41598-025-95946-3 PMC12003728

[41] Hu B, et al. Proteomic analysis of the faba bean-wheat intercropping system in controlling the occurrence of faba bean fusarium wilt due to stress caused by Fusarium oxysporum f. sp. fabae and benzoic acid. BMC Plant Biol. 2023;23(1):472. 37803265 10.1186/s12870-023-04481-8 PMC10557263

[42] Liu J, et al. TMEM164 is a new determinant of autophagy-dependent ferroptosis. Autophagy. 2023;19(3):945–56. 35947500 10.1080/15548627.2022.2111635 PMC9980451

[43] Huang Y et al. Lactylation stabilizes TFEB to elevate autophagy and lysosomal activity. J Cell Biol. 2024;223(11). 10.1083/jcb.202308099 PMC11354204 39196068

[44] Szabo V et al. Orai1 calcium channel inhibition prevents progression of chronic pancreatitis. JCI Insight. 2023;8(13). 10.1172/jci.insight.167645 PMC10371343 37227782

[45] Ubaid S, et al. Comprehensive analysis of oncogenic determinants across tumor types via multi-omics integration. Cancer Genet. 2025;298–9:44–62. 10.1016/j.cancergen.2025.08.010 40914134

[46] Baran A. A study to assess the pharmacological agent potential of gold nanoparticles and their effects on human cancer cells and hospital pathogens using in vitro methods. Front Pharmacol. 2024;15:1498734. 39834814 10.3389/fphar.2024.1498734 PMC11743435

[47] Nimhan G, Narwade M, Gajbhiye K. Biosensor driven biomarker analysis: pioneering advancements in cancer diagnosis and therapeutic strategies. Biomarkers. 2025;30(4):332–51. 40476629 10.1080/1354750X.2025.2515363

