# Supplementary figures and images for: Investigating the role of lncRNA SNHG14 in early diagnosis and prognosis of acute pancreatitis: a bioinformatics exploration
Source: Hereditas. 2026 Mar 11;163:52. doi: 10.1186/s41065-026-00656-z (PMC13088405; doi:10.1186/s41065-026-00656-z)

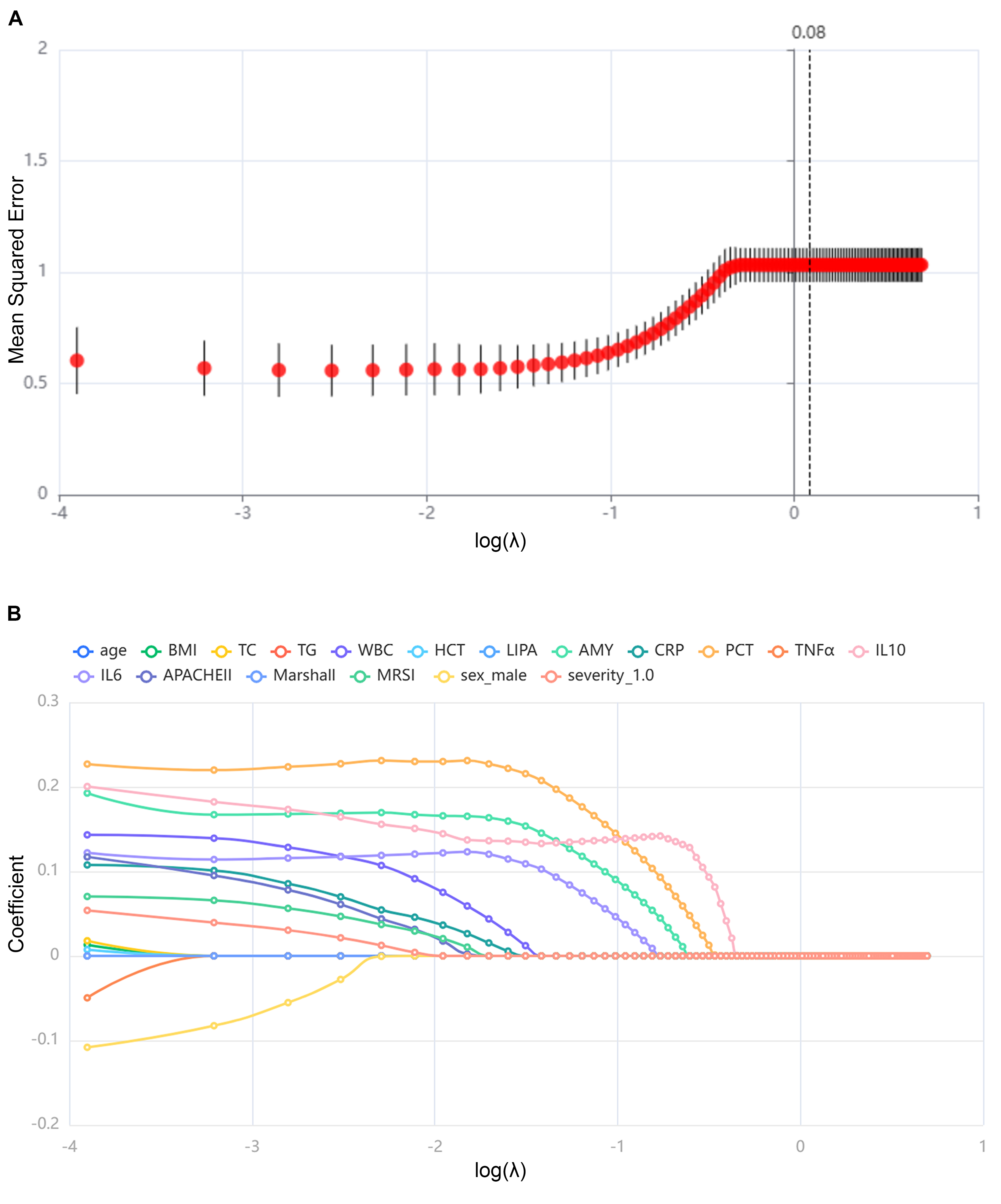

Supplement: Supplementary file 1 — Supplementary Material 1: Figure S1. Least absolute shrinkage and selection operator (LASSO) regression analysis for variable selection in the Cox model. A, Cross-validation curve (λ=0.081). B, Coefficient path plot. [file 41065_2026_656_MOESM1_ESM.tif]
