# Supplementary material for: Investigating the role of lncRNA SNHG14 in early diagnosis and prognosis of acute pancreatitis: a bioinformatics exploration
Source: Hereditas. 2026 Mar 11;163:52. doi: 10.1186/s41065-026-00656-z (PMC13088405; doi:10.1186/s41065-026-00656-z)
Supplement: Supplementary file 2 — Supplementary Material 2. [file 41065_2026_656_MOESM2_ESM.docx]

**Supplementary Table 1.** Primer sequences for qRT-PCR and cell transfection.

| Genes | Sequences (5’-3’) |
| --- | --- |
| SNHG14 | F: CGTTGTCGAAAGCTAAAAGGA |
|  | R: TGTTTCCATCTCACCAAATGC |
| miR-30a-5p | F: UGUAAACAUCCUCGACUGGAAG |
|  | R: TTGGCAGGTACAAACCAAAGAT |
| GAPDH | F: GCACCGTCAAGGCTGAGAAC |
|  | R: TGGTGAAGACGCCAGTGGA |
| U6 | F: CTCGCTTCGGCAGCACA |
|  | R: AACGCTTCACGAATTTGCGT |
| si-SNHG14 | GCAAAUGAAAGCUACCAAU |
| si-NC | UUCUCCGAACGUGUCACGUTT |
| miR-30a-5p inhibitor | CUUCCAGUCGAGGAUGUUUA |
| Inhibitor NC | CAGUACUUUUGUGUAGUACAA |
