# Supplementary material for: Investigating the role of lncRNA SNHG14 in early diagnosis and prognosis of acute pancreatitis: a bioinformatics exploration
Source: Hereditas. 2026 Mar 11;163:52. doi: 10.1186/s41065-026-00656-z (PMC13088405; doi:10.1186/s41065-026-00656-z)
Supplement: Supplementary file 3 — Supplementary Material 3. [file 41065_2026_656_MOESM3_ESM.docx]

**Supplementary Table 2**. DeLong’s test results for the comparison of AUCs.

|  | z | *P* | AUC difference | SE | 95% CI |
| --- | --- | --- | --- | --- | --- |
| APACHEII - SNHG14 | 0.328 | 0.743 | 0.019 | 0.291 | (-0.095 - 0.133) |
| Marshall - SNHG14 | 0.169 | 0.866 | 0.010 | 0.298 | (-0.107 - 0.127) |
| MRSI - SNHG14 | -0.121 | 0.904 | -0.007 | 0.297 | (-0.122 - 0.108) |
| SNHG14 - Combined | -2.780 | 0.005 | -0.115 | 0.268 | (-0.196 - -0.034) |

**Notes:** AUC, area under the curve; SE, standard error; 95% CI, 95% confidence interval.
