# Supplementary material for: Investigating the role of lncRNA SNHG14 in early diagnosis and prognosis of acute pancreatitis: a bioinformatics exploration
Source: Hereditas. 2026 Mar 11;163:52. doi: 10.1186/s41065-026-00656-z (PMC13088405; doi:10.1186/s41065-026-00656-z)
Supplement: Supplementary file 4 — Supplementary Material 4. [file 41065_2026_656_MOESM4_ESM.docx]

**Supplementary Table 3.** Parameters used in the multivariate logistic regression analysis for developing a diagnostic model.

|  | B | S.E. | Wald | df | *P* | Exp (B) | 95% Cl for EXP(B) | |
| --- | --- | --- | --- | --- | --- | --- | --- | --- |
|  |  |  |  |  |  |  | Lower | Upper |
| APACHE II | 0.245 | 0.097 | 6.328 | 1 | 0.012 | 1.278 | 1.056 | 1.547 |
| Marshall | 0.582 | 0.213 | 7.422 | 1 | 0.006 | 1.789 | 1.177 | 2.718 |
| MRSI | 0.272 | 0.114 | 5.661 | 1 | 0.017 | 1.312 | 1.049 | 1.642 |
| SNHG14 | 1.596 | 0.803 | 3.948 | 1 | 0.047 | 4.936 | 1.022 | 23.838 |
| Constant | -8.140 | 1.553 | 27.467 | 1 | 0.000 | 0.000 |  |  |

Diagnostic model for the SAP was established by logistic regression analysis according to APACHE II score, modified Marshall score, MRSI, and SNHG14. They were independently related to the severity of AP. Based on the parameters of logistic regression analysis, the SAP diagnostic probability regression model was established:

| P_SAP_= | e^-8.140+0.245APACHE II+0.582Marshall+0.272MRSI+1.596SNHG14^ |
| --- | --- |
|  | 1＋e^-8.140+0.245APACHE II+0.582Marshall+0.272MRSI+1.596SNHG14^ |
